# Supplementary material for: Access to hypertension services and health-seeking experiences in rural Coastal Kenya: A qualitative study
Source: PLOS Glob Public Health. 2025 Nov 21;5(11):e0004324. doi: 10.1371/journal.pgph.0004324 (PMC12637890; doi:10.1371/journal.pgph.0004324)
Supplement: S1 File — (PDF) [file pgph.0004324.s001.pdf]

Access to hypertension services and health-seeking experiences in rural Coastal Kenya: A qualitative study: SRQR Checklist[1]

Topic	No.	Item	Page No
**Title**	S1	Concise description of the nature and topic of the study Identifying the study as qualitative or indicating the approach (e.g., ethnography, grounded theory) or data collection methods (e.g., interview, focus group) is recommended	Pg. 1, Line 1-2
**Abstract**	S2	Summary of key elements of the study using the abstract format of the intended publication; typically includes background, purpose, methods, results, and conclusions	Pg. 2, Line 31-64
**Introduction**			
Problem formulation	S3	Description and significance of the problem/phenomenon studied; review of relevant theory and empirical work; problem statement	Pg.4-6, Line 71-136
Purpose or research question	S4	Purpose of the study and specific objectives or questions	Pg. 6, Line 119-136
**Methods**			
Qualitative approach and research paradigm	S5	Qualitative approach (e.g., ethnography, grounded theory, case study, phenomenology, narrative research) and guiding theory if appropriate; identifying the research paradigm (e.g., postpositivist, constructivist/ interpretivist) is also recommended; rationale	Pg.7-9, Line 152-202
Researcher characteristics and reflexivity	S6	Researchers' characteristics that may influence the research, including personal attributes, qualifications/experience, relationship with participants, assumptions, and/or presuppositions; potential or actual interaction between researchers' characteristics and the research questions, approach, methods, results, and/or transferability	Pg. 11, Line 239-242
Context	S7	Setting/site and salient contextual factors; rationale	Pg.9, Line 206-222
Sampling strategy	S8	How and why research participants, documents, or events were selected; criteria for deciding when no further sampling was necessary (e.g., sampling saturation); rationale	Page 11; Line 230-245
Ethical issues pertaining to human subjects	S9	Documentation of approval by an appropriate ethics review board and participant consent, or explanation for lack thereof; other confidentiality and data security issues	Pg. 13, Line 296-302

Data collection methods	S10	Types of data collected; details of data collection procedures including (as appropriate) start and stop dates of data collection and analysis, iterative process, triangulation of sources/methods, and modification of procedures in response to evolving study findings; rationale	Pg. 11-12; Line 240-258
Data collection instruments and technologies	S11	Description of instruments (e.g., interview guides, questionnaires) and devices (e.g., audio recorders) used for data collection; if/how the instrument(s) changed over the course of the study	Pg.11-12, Line 248-266
Units of study	S12	Number and relevant characteristics of participants, documents, or events included in the study; level of participation (could be reported in results)	Pg.15-16, Line 305-312
Data processing	S13	Methods for processing data prior to and during analysis, including transcription, data entry, data management and security, verification of data integrity, data coding, and anonymization/deidentification of excerpts	Pg. 12, Line 261-264
Data analysis	S14	Process by which inferences, themes, etc., were identified and developed, including the researchers involved in data analysis; usually references a specific paradigm or approach; rationale	Pg. 12-13, Line 269-293
Techniques to enhance trustworthiness	S15	Techniques to enhance trustworthiness and credibility of data analysis (e.g., member checking, audit trail, triangulation); rationale	Pg. 13, Line 286-293
**Results**			
Synthesis and interpretation	S16	Main findings (e.g., interpretations, inferences, and themes); might include development of a theory or model, or integration with prior research or theory	Pg. 16-28, Line 316-637
Links to empirical data	S17	Evidence (e.g., quotes, field notes, text excerpts, photographs) to substantiate analytic findings	Pg. 16-29, Line 316-637
**Discussion**			
Integration with prior work, implications, transferability, and contribution(s) to the field	S18	Short summary of main findings; explanation of how findings and conclusions connect to, support, elaborate on, or challenge conclusions of earlier scholarship; discussion of scope of application/ generalizability; identification of unique contribution(s) to scholarship in a discipline or field	Pg. 30-35, Line 641-771;

Limitations	S19	Trustworthiness and limitations of findings	Pg. 35, Line 773-784
**Other**			
Conflicts of interest	S20	Potential sources of influence or perceived influence on study conduct and conclusions; how these were managed	Pg. 37, Line 823
Funding	S21	Sources of funding and other support; role of funders in data collection, interpretation, and reporting	Pg. 36, Line 810-815

Reference

[1] O'Brien BC, Harris IB, Beckman TJ, Reed DA, Cook DA. Standards for reporting qualitative research: a synthesis of recommendations. Acad Med. 2014;89(9):1245-1251.
